# Induction of Cell Death in Growing Human T-Cells and Cell Survival in Resting Cells in Response to the Human T-Cell Leukemia Virus Type 1 Tax

**DOI:** 10.1371/journal.pone.0148217

**Published:** 2016-02-01

**Authors:** Mariko Mizuguchi, Yuka Sasaki, Toshifumi Hara, Masaya Higuchi, Yuetsu Tanaka, Noriko Funato, Nobuyuki Tanaka, Masahiro Fujii, Masataka Nakamura

**Affiliations:** 1 Human Gene Sciences Center, Tokyo Medical and Dental University, Tokyo, Japan; 2 Division of Virology, Niigata University Graduate School of Medical and Dental Sciences, Niigata, Japan; 3 Department of Immunology, Graduate School and Faculty of Medicine, University of the Ryukyus, Okinawa, Japan; 4 Division of Cancer Biology and Therapeutics, Miyagi Cancer Center Research Institute, Miyagi, Japan; Johns Hopkins School of Medicine, UNITED STATES

## Abstract

Tax1 encoded by the human T-cell leukemia virus type 1 (HTLV-1) has been believed to dysregulate the expression of cellular genes involved in cell survival and mortality, leading to the development of adult T-cell leukemia (ATL). The function of Tax1 in ATL development however is still controversial, primarily because Tax1 induces cell cycle progression and apoptosis. To systemically understand cell growth phase-dependent induction of cell survival or cell death by Tax1, we established a single experimental system using an interleukin 2 (IL-2)-dependent human T-cell line Kit 225 that can be forced into resting phase by IL-2 deprivation. Introduction of Tax1 and HTLV-2 Tax (Tax2B) decreased mitochondrial activity alongside apoptosis in growing cells but not in resting cells. Cell cycle profile analysis indicated that Tax1 and Tax2B were likely to perturb the S phase in growing cells. Studies with Tax1 mutants and siRNA for NF-κB/RelA revealed that Tax1-mediated cell growth inhibition and apoptosis in growing Kit 225 cells depend on RelA. Interestingly, inactivation of the non-canonical NF-κB and p38 MAPK pathways relieved Tax1-mediated apoptosis, suggesting that the Tax1-NF-κB-p38 MAPK axis may be associated with apoptosis in growing cells. Inflammatory mediators such as CCL3 and CCL4, which are involved in oncogene-induced senescence (OIS), were induced by Tax1 and Tax2B in growing cells. In contrast, RelA silencing in resting cells reduced mitochondrial activity, indicating that NF-κB/RelA is also critical for Tax1-mediated cell survival. These findings suggest that Tax1-mediated cell survival and death depend on the cell growth phase. Both effects of Tax1 may be implicated in the long latency of HTLV-1 infection.

## Introduction

Human T-cell leukemia virus type 1 (HTLV-1), a human oncogenic retrovirus, is the causative agent of an aggressive CD4^+^ T-cell malignancy, adult T-cell leukemia/lymphoma (ATL/ATLL) [1–3] and HTLV-1-associated myelopathy/tropical spastic paraparesis (HAM/TSP) [4, 5]. Approximately 2–5% of HTLV-1-infected individuals develop ATL after a long latent period. The mechanisms underlying the development of ATL, however, are incompletely understood. HTLV-1 encodes the oncogenic protein Tax1 that is believed to be implicated in cellular immortalization and clonal expansion at the incipient stages of ATL development. Tax1 dysregulates the expression of cellular genes involved in physiological processes of cell growth, survival and mortality through at least three transcriptional factors, nuclear factor (NF)-κB, cAMP response element-binding protein (CREB) and serum response factor (SRF) [6]. Disturbance of the intracellular environment by Tax1 is considered critical for cell immortalization and transformation.

Abnormal cell cycle progression is potential for cellular transformation. Cell cycle progression is tightly regulated by complexes of cyclins and cyclin-dependent kinases (CDK). Most somatic cells remain at the G0/G1 phase. G1 cyclin-CDK complexes activated by mitogenic stimulation phosphorylate the retinoblastoma tumor suppressor protein (pRB), leading to the release of active E2F, which further regulates the transcription of genes involved in cell cycle progression and DNA replication [7–9]. Tax1 has been previously reported to induce G1 cyclin-CDK complexes, including cyclin D2, CDK4 and CDK2, thereby causing E2F activation [10–12]. Tax1 expression aids in cell cycle progression from the G0/G1 phase to the S phase in resting-induced lymphocytes without any mitogenic stimulation [10–13]. Tax1 thus plays an important role in abnormal cell cycle progression.

Apoptosis is an important process to eliminate uncontrolled and abnormal cells via multiple network signaling pathways such as sequential caspase cascade and Bcl-2 family proteins [14, 15]. Cellular mortality is determined by maintaining a balance between pro- and anti-apoptosis molecules. Most cancer cells acquire resistance to apoptosis. Tax1 activates the caspase inhibitor survivin and X-linked inhibitor of apoptosis protein (XIAP), and the Bcl-2 family protein Bcl-xL, leading to cell survival [16–18]. Tax1 expression is also shown to prevent apoptosis by serum starvation and treatment with topoisomerase inhibitor in Jurkat cells [19]. Prevention of apoptosis by Tax1 may be associated with the accumulation of abnormal cells.

In contrast to Tax1-dependent cell cycle progression and cell survival, previous studies have also shown that Tax1 expression induces cell growth inhibition and apoptosis [20, 21]. Gene expression profiles show that Tax1 modulates both cell survival- and apoptosis-related genes in HTLV-1-infected Tax1-expressing T-cells (C81) and HeLa cells [22, 23]. Cell growth inhibition is induced at least in part by the CDK inhibitors p21 and p27, which are up-regulated by Tax1 [19, 24, 25]. In Jurkat cells, Tax1 induces apoptosis, presumably through the expression of tumor necrosis factor (TNF) family-related death ligands, TNF-related apoptosis-inducing ligand (TRAIL) and FasL [20, 26]. C81 cells showed increased in sensitivity to apoptosis induced by DNA damage agents [22].

The delta-retrovirus HTLV-2 is close to HTLV-1 in terms of genome sequence and gene functions. HTLV-2 however has not been shown to be certainly associated with the development of hematological malignant diseases despite its ability to immortalize *in vitro* human T-cells in an interleukin (IL)-2-dependent manner [27]. HTLV-2 codes for Tax2, which transcriptionally activates the NF-κB and CREB pathways, similar to Tax1 [28, 29]. Tax2 also induces cell cycle progression and prevents apoptosis in serum-starved Jurkat cells [19]. Little is known about the relation between Tax2 and induction of apoptosis.

In this study, we re-evaluated the induction of cell survival or death by Tax1 in the same cells, and found that Tax1 mediated cell survival and apoptosis in resting and growing cells, respectively, in an NF-κB/RelA-dependent manner. These observations imply that the pleiotropic effects of Tax1 may be associated with the latency of HTLV-1.

## Materials and Methods

### Cells and cell culture

The IL-2 dependent human T-cell line Kit 225 was maintained in RPMI 1640 medium containing 10% fetal calf serum (FCS) and 1 nM IL-2 [30]. The human acute lymphocytic leukemia T-cell line Jurkat and its derivative JPX-9 were maintained in RPMI 1640 medium containing 10% FCS [31, 32]. JPX-9 carries the Tax1 gene under the control of the metallothionein promoter. In JPX-9 cells, Tax1 expression was induced by the addition of 20 μM CdCl_2_. Peripheral blood lymphocytes (PBLs) were obtained from healthy adults by using Ficoll-Paque PLUS (GE Healthcare, Piscataway, NJ, USA), with approval from the Internal Review Committee of Tokyo Medical and Dental University (Permit Number: 1053). We enrolled participants into the study after obtaining written informed consent. PBLs were cultured in RPMI 1640 medium containing 20% FCS with 10 μg/ml phytohemagglutinin for 72 h, washed with serum-free RPMI 1640 medium, and maintained in RPMI 1640 medium containing 20% FCS for 48 h.

### Plasmid construction

Expression vectors based on the human β-actin promoter for Tax1 (pMT-2Tax), its mutants (TaxM22, Taxd3 and Tax703) and Tax2B (pHβAP-r-1-neoTax2B) have been previously described [33, 34]. pHβAP-r-1-neo was used as a control plasmid [35]. The luciferase reporter plasmids pGL3/E2WTx4-Luc, pGL3/E2MTx4-Luc and pGL3/κB-Luc have been described elsewhere [36]. The PathDetect reporter system (Agilent Technologies, Santa Clara, CA, USA) consists of the luciferase reporter plasmid (pFR-Luc) and the fusion transcription factor expression plasmids (pFA-CHOP and pFA2-c-Jun). pFR-Luc carries a DNA element to bind the phosphorylated forms of the fusion proteins, GAL4-dbd-CHOP and GAL4-dbd-c-Jun, which are activated by the phosphorylation with p38 and JNK, respectively.

### Transfection and reporter assay

The expression and reporter plasmids were introduced into asynchronously growing Kit 225 cells by the DEAE-dextran method as described previously [37]. The cells were cultured for 48 or 72 h with or without IL-2, and luciferase activity was measured using the Luciferase Assay System (Promega, Madison, WI, USA), according to the manufacturer’s protocol. Luciferase activity was normalized relative to the protein concentration measured by using the DC Protein Assay Kit (Bio-Rad, Hercules, CA, USA). All assays were performed at least three times in duplicate, and the values are presented as means ± SE.

### RNA isolation and quantitative PCR (qPCR)

Total RNA was extracted using Isogen (Nippon Gene, Toyama, Japan), according to the manufacturer’s protocol. First-strand cDNA was synthesized using the 1st Strand cDNA Synthesis Kit for RT-PCR (AMV) (Roche Applied Science, Indianapolis, IN, USA) according to the supplier’s protocol. Quantitative detection of mRNA was performed by qPCR in a LightCycler (Roche). The PCR primer sequences are shown in S1 Table. The primers for 18 S rRNA were obtained from TaKaRa.

### Infection with recombinant adenoviruses

Recombinant adenoviruses for Tax1 (Ad-Tax1) and Tax2B (Ad-Tax2B) were described elsewhere [38]. The adenoviruses for Tax1 mutants, Ad-TaxM22, Ad-Tax703, Ad-Taxd17/5 and Ad-Tax225-232 were generated using the ViraPower™ adenoviral expression system (Invitrogen Life Technologies, Grand Island, NY, USA). Cells were infected with Tax-expressing adenoviruses or control virus (Ad-Con) at multiples of 100 plaque-forming units (PFU)/cell for Kit 225 and PBLs, and 10 PFU/cell for Jurkat cells as described previously [11, 39].

### MTT and LDH assays

Cells in 500 μl of culture were added with 50 μl of 5 mg/ml 3-(4,5-dimethylthiazol-2-yl)-2, 5-diphenyltetrazolium bromide (MTT) (Sigma, St. Louis, MO, USA) solution and incubated for 4 h. Living cells convert yellow MTT to dark-blue formazan crystals. The crystals were dissolved in the same volume of acidified isopropanol. The absorption of the resulting solution was checked at 570 nm by using a spectrophotometer (Bio-Rad). Lactate dehydrogenase (LDH) released in the culture medium was measured using the Cytotoxicity Detection Kit (Roche), according to the supplier’s recommendation.

### DNA content analysis

Cells were stored in 70% ethanol at -20°C. DNA was stained with PI/RNase staining buffer (BD Pharmingen, San Diego, CA, USA), according to the manufacturer’s protocol. Cells were analyzed by flow cytometry (FACSCalibur, BD Biosciences, San Jose, CA, USA).

### Detection of DNA fragmentation (TUNEL assay) and Tax1 expression

Apoptosis was determined using APO-BRDU TUNEL Assay Kit (Nippon Gene), according to the supplier’s protocol. Cells were harvested 72 h after adenovirus infection, washed with phosphate-buffered saline containing 0.2% bovine serum albumin, and stained with biotin-conjugated anti-Tax1 monoclonal antibody (Lt-4) with streptavidin-phycoerythrin (PE) (BD Pharmingen). Cells were analyzed by flow cytometry.

### Detection of Annexin V-positive cells

Cells were stained with Muse Annexin V and Dead Cell Kit (Merck Millipore, Darmstadt, Germany), according to the supplier’s protocol. Annexin V-positive cells were counted using Muse Cell Analyzer (Merck Millipore).

### Small interfering RNA (siRNA)

The siRNAs for RelA (168182G07, 168182G09 and 168182G11), p100 (202722G03, 201738F04 and 201738E12), TRAIL (287134E03, 287134E05 and 287134E07) and control siRNA were purchased from Invitrogen. siRNA was introduced using the Neon Transfection System (Invitrogen Life Technologies) a day before adenovirus infection.

### Immunoblotting analysis

Cells were lysed in the RIPA buffer containing 150 mM NaCl, 50 mM Tris-HCl (pH 8.0), 1% Nonidet P-40, 0.5% deoxycholate and 0.1% sodium dodecyl sulfate (SDS). Nuclear and cytoplasmic extracts were prepared using the NE-PER Nuclear and Cytoplasmic Extraction Reagents (Thermo Scientific, Waltham, MA, USA). Cell extracts were separated by SDS-polyacrylamide gel electrophoresis (SDS-PAGE) and blotted onto Immobilon-P membranes (Atto, Tokyo, Japan). The membranes were soaked in 5% skim milk solution and incubated with antibodies. Then, these membranes were incubated with horseradish peroxidase-conjugated secondary antibodies (GE Healthcare), and the proteins were detected using the Luminata Crescendo Western HRP Substrate (Merck Millipore), according to the manufacturer’s protocol.

### Antibodies

The anti-Tax1 antibodies Lt-4 and Taxy-7 and the anti-Tax2 (GP3738) antibody have been previously described [40–42]. Anti-RelA (sc-372), anti-p52 (sc-3786), anti-p38α (sc-535), anti-JNK (sc-571), anti-nucleolin (sc-13057) and β-tubulin (sc-9104) antibodies were obtained from Santa Cruz Biotechnology (Santa Cruz, CA, USA). Anti-phospho-p38 (#9211), anti-phospho-SAPK/JNK (#9251), anti-phospho-Erk1/2 (#9106) and anti-Erk1/2 (#9102) antibodies were purchased from Cell Signaling (Danvers, MA, USA).

### Statistical analysis

A paired *t* test was performed for statistical analysis. *p* values less than 0.05 were considered significant.

## Results

### Tax1 and Tax2B disrupt cell cycle status in growing cells

**Fig 1:**
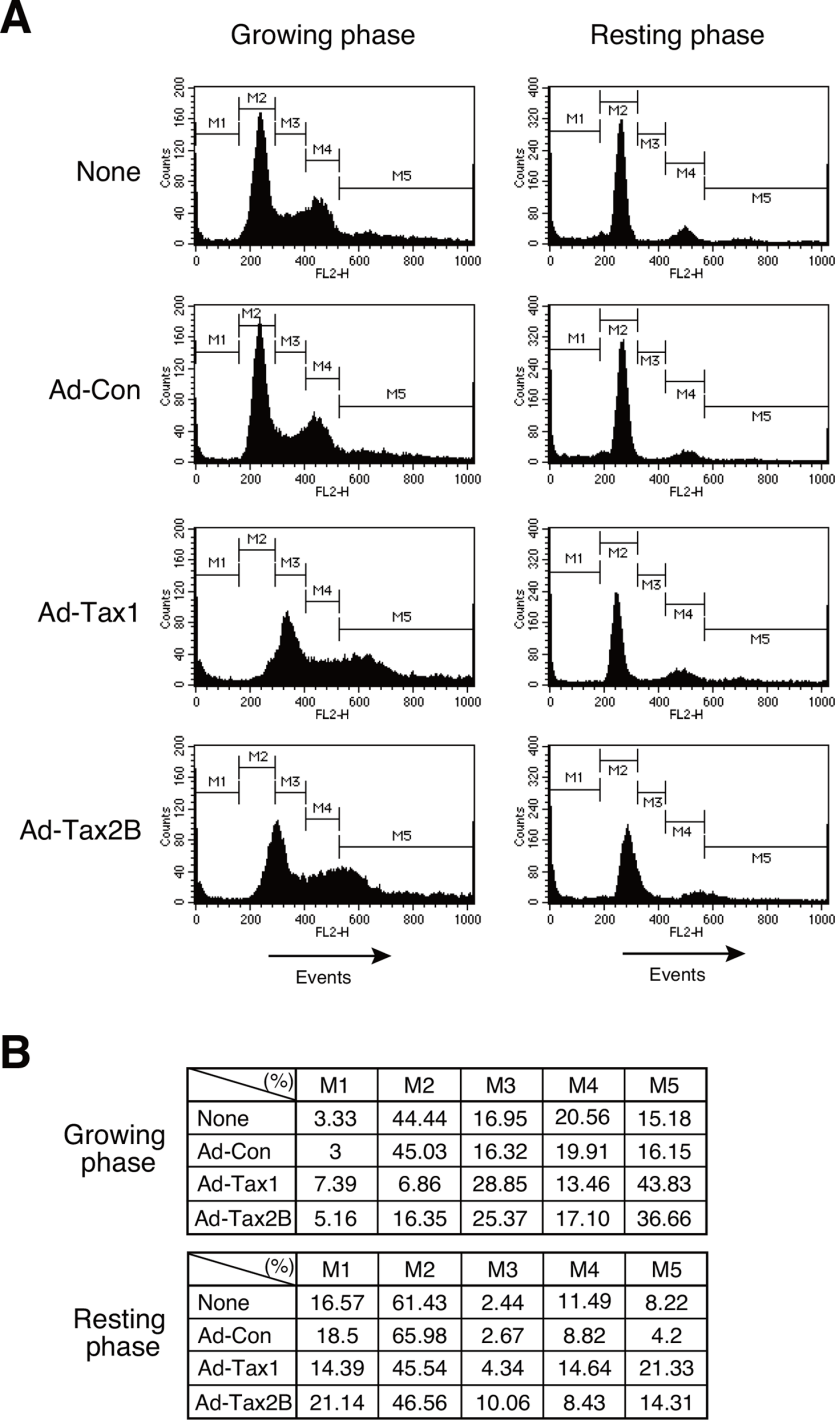
Cell cycle profiles of growing and resting cells with Tax1. (A) Growing or resting Kit 225 cells were infected with recombinant adenoviruses expressing Tax1 (Ad-Tax1), Tax2B (Ad-Tax2B) or control virus (Ad-Con), and cultured for 72 h. DNA content was determined using a flow cytometer after PI staining. (B) Percentages of cell cycle phases were calculated from Fig 1A. M1, M2, M3, M4 and M5 indicate the sub-G0, G0/G1, S, G2/M and multinuclear cells, respectively.

Tax1 has previously been shown to induce cell cycle progression from the G0/G1 phase to the S phase in resting-induced PBLs and Kit 225 cells [10–13]. In contrast, it has also been reported to induce apoptosis in Jurkat cells [20, 26]. This completely opposing phenotypic function of Tax1 is quite intriguing. To assess this issue, growing and resting Kit 225 cells were examined for their response to Tax1 or Tax2B expression by recombinant adenovirus infection. Kit 225 cells offer an advantage: cell culture in IL-2-depleted medium for 48 h causes approximately 65% of the cells to become quiescent, and the addition of IL-2 proceeds the cell cycle (Fig 1A) [11]. We investigated the cell cycle profiles of respective cells based on the DNA content, as determined by flow cytometry. At 72 h after recombinant adenovirus infection, growing cells with Tax1 and Tax2B showed a drastic increase in the cell population with more than 4C DNA content in association with a significant increase in the S phase and decrease in the G0/G1 and G2/M phases (Fig 1A and 1B). Upon infection with Tax1 and Tax2B recombinant adenoviruses, resting cells showed a decrease in the G0/G1 phase and increase in the cells population with more than 4C DNA content (Fig 1A and 1B). Infection with the control adenovirus did not alter cell cycle profiles, relative to the uninfected controls, in both growing and resting cells. These results suggest that Tax1 and Tax2B cause dysfunctional DNA replication at the S phase in growing cells.

### Tax1 and Tax2B induce growth inhibition in growing cells

**Fig 2:**
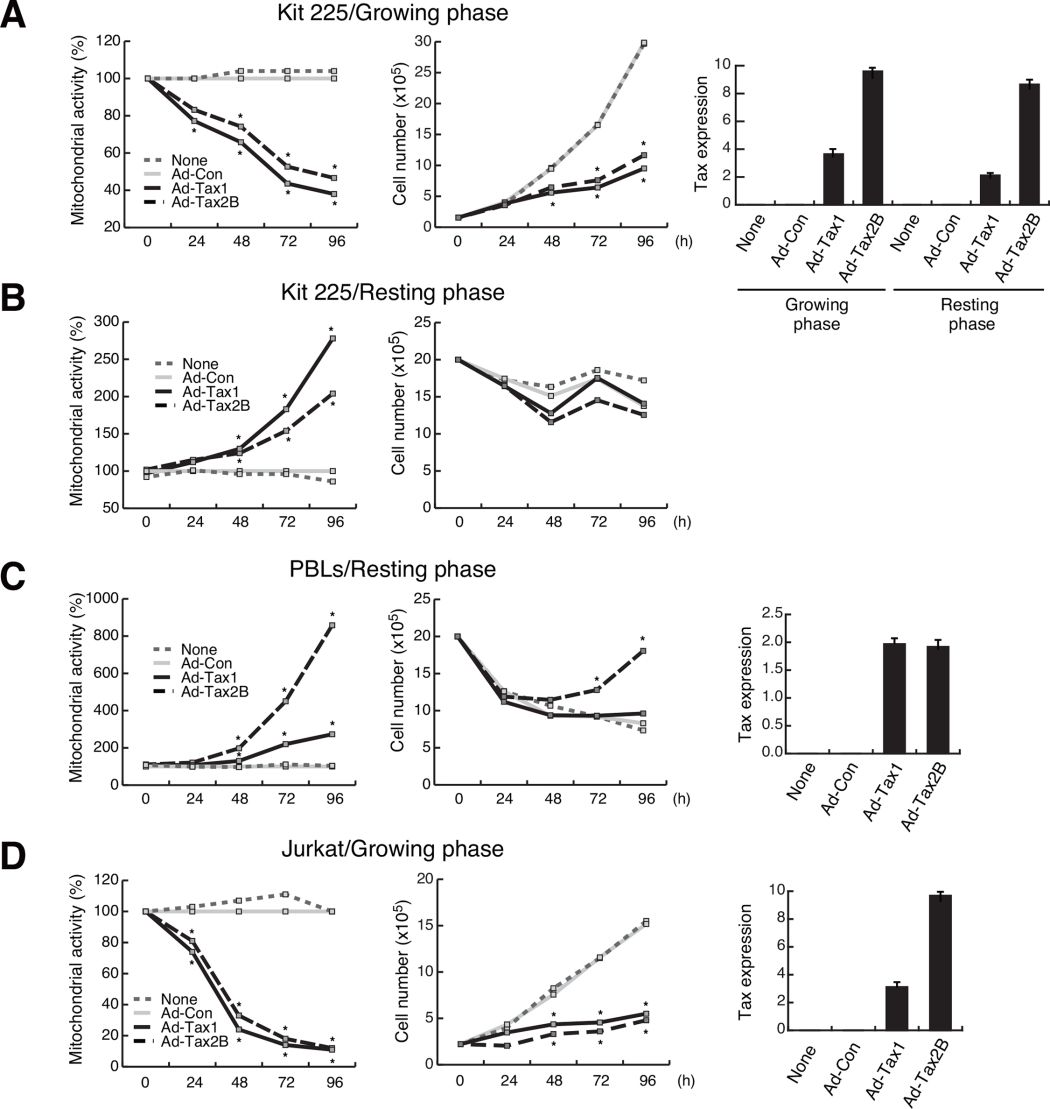
Differential effects of Tax1 on cell proliferation. Growing (A) or resting (B) Kit 225 cells, PBLs (C) and Jurkat cells (D) were infected with Ad-Tax1, Ad-Tax2B or Ad-Con, and cultured for the indicated times. Mitochondrial activity was measured by the MTT assay and the cells were enumerated. Relative percentages of Ad-Tax1 or Ad-Tax2B samples to Ad-Con samples are shown. *, *p* < 0.05. Tax expression was monitored by qPCR. Values are shown as the means of the copy numbers ± SE after normalization against 18 S rRNA content.

To further pursue the different effects of Tax1 and Tax2B between growing and resting Kit 225 cells, intracellular mitochondrial enzyme activity was measured by the MTT assay to monitor cellular activity. Upon expression of Tax1 or Tax2B, Kit 225 cells in the growing phase showed slower increase in cell number with reduction of mitochondrial activity, compared to Ad-Con-infected cells (Fig 2A). In resting cells, Tax1 and Tax2B significantly increased mitochondrial activity, while no or little, if any, increase in cell number was observed (Fig 2B). Tax1 and Tax2B increased the mitochondrial activity in resting-induced PBLs. In particular, Tax2B profoundly increased the activity, which was different from observation in Kit 225 cells infected with Tax2B adenovirus (Fig 2C). The difference can be attributed to the Tax2B function that IL-2 was profoundly produced in PBLs [38], while Kit 225 cells did not generate any appreciable amounts of IL-2 (data not shown). Thus, Tax2B is likely to continuously support cell growth in PBLs culture (Fig 2C). Autonomously growing Jurkat cells showed the reduction of mitochondrial activity and slower cell growth in response to Tax1 and Tax2B (Fig 2D). These results suggest that the cell cycle phase critically influences the effects of Tax1 on the fate of human T-cells.

### Tax1 activates cell cycle-related genes in resting cells

**Fig 3:**
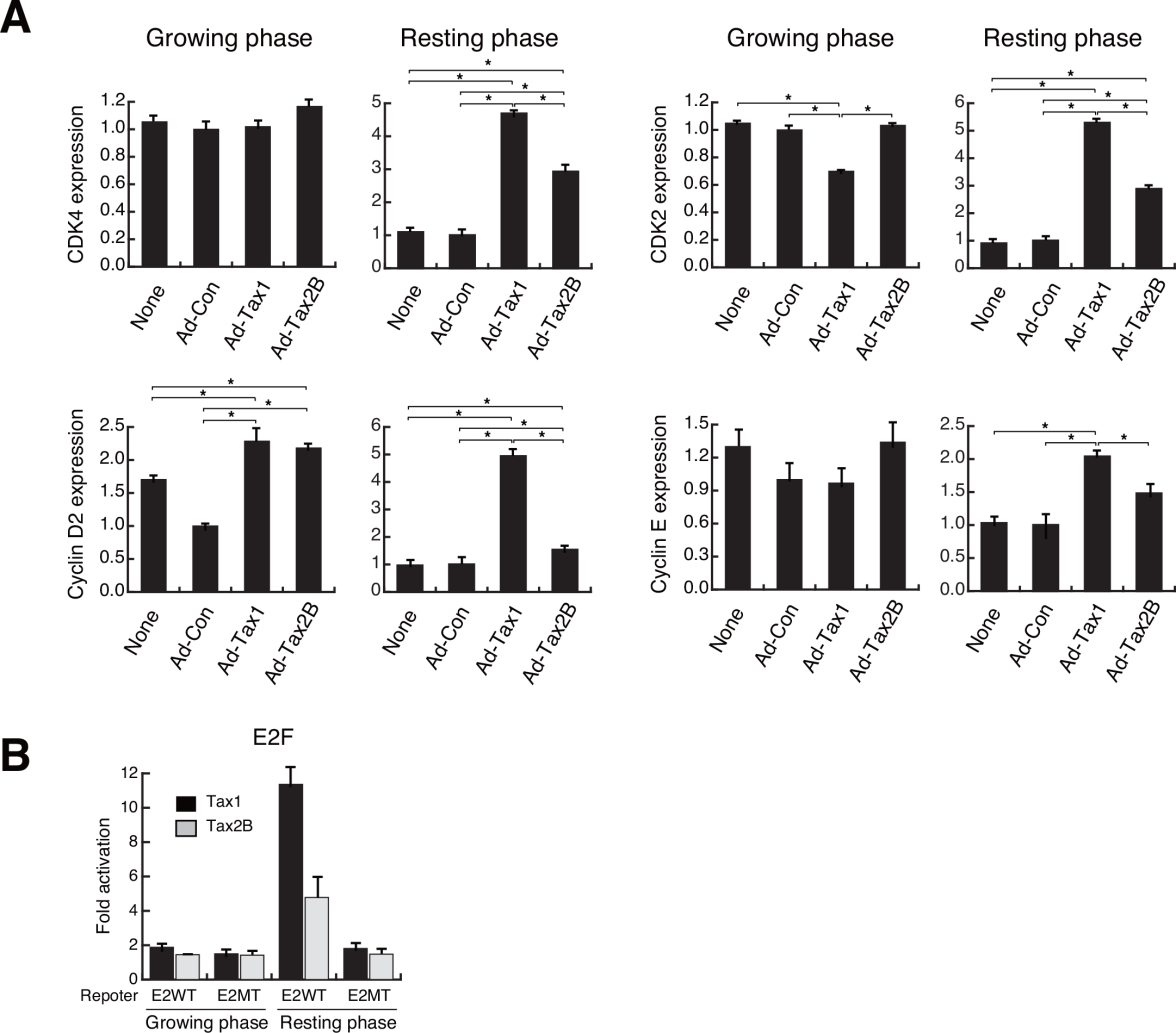
Induction of cell cycle regulatory genes by Tax1 in resting cells. (A) Growing or resting Kit 225 cells were infected with Ad-Tax1 or Ad-Tax2B. Cells were cultured for 72 h and harvested for RNA isolation. The levels of CDK4, cyclin D2, CDK2 and cyclin E mRNA were measured by qPCR. Values are shown as the means ± SE after normalization against 18 S rRNA content. *, *p* < 0.05. (B) Reporter plasmids carrying binding sites for the wild-type and point-mutated E2F were transfected into Kit 225 cells along with Tax1 and Tax2B expression plasmids (pMT-2Tax and pHβAP-r-1-neoTax2B, respectively). After 48 h of culture with or without IL-2, the cells were harvested for luciferase assay, which was subsequently normalized against protein content. Values are shown as fold activation relative to luciferase activity of cells with pHβAP-r-1-neo (means ± SE).

Tax1 has been shown to activate a set of cell cycle regulatory genes, leading to cell cycle progression [10, 13]. To confirm cell growth-dependent activation of genes for the cell cycle regulatory molecules included in the G1 cyclin-CDK complexes, we studied the expression of CDK4, cyclin D2, CDK2 and cyclin E genes in Kit 225 cells with or without Tax1 by qPCR. Introduction of Tax1 elevated the mRNA levels of CDK4, cyclin D2, CDK2 and cyclin E genes in resting cells (Fig 3A). Tax2B up-regulated the mRNA levels of those genes in resting cells, although the increase in the cyclin E mRNA level was not statistically significant (Fig 3A). In growing cells, Tax1 and Tax2B increased cyclin D2 mRNA level (Fig 3A). Interestingly, CDK2 expression was down-regulated by Tax1 in growing cells (Fig 3A). Cyclin E expression paralleled that of CDK2, which is a partner of cyclin E, although statistical analysis did not show significance in the case of growing cells (Fig 3A).

The G1 cyclin-CDK complexes phosphorylate the retinoblastoma tumor suppressor protein (Rb), releasing and activating the transcriptional factor E2F that regulates genes involved in cell cycle progression [11, 43]. We thus examined the effects of Tax1 and Tax2B on E2F activation. Reporter assays with the wild-type or mutant E2F-binding site showed that Tax1 significantly activated the wild-type E2F-binding site in resting cells, and that similar activation was induced by Tax2B, but to a lesser extent (Fig 3B). Growing cells did not respond to Tax1 and Tax2B in terms of E2F activation (Fig 3B). A substitute mutant of the E2F-binding site was not activated by Tax1 or Tax2B in resting and growing cells (Fig 3B). These results indicate that Tax1 efficiently activates E2F in resting cells, presumably resulting in the induction of cell cycle-related genes.

### Tax1 induces apoptosis in growing cells

**Fig 4:**
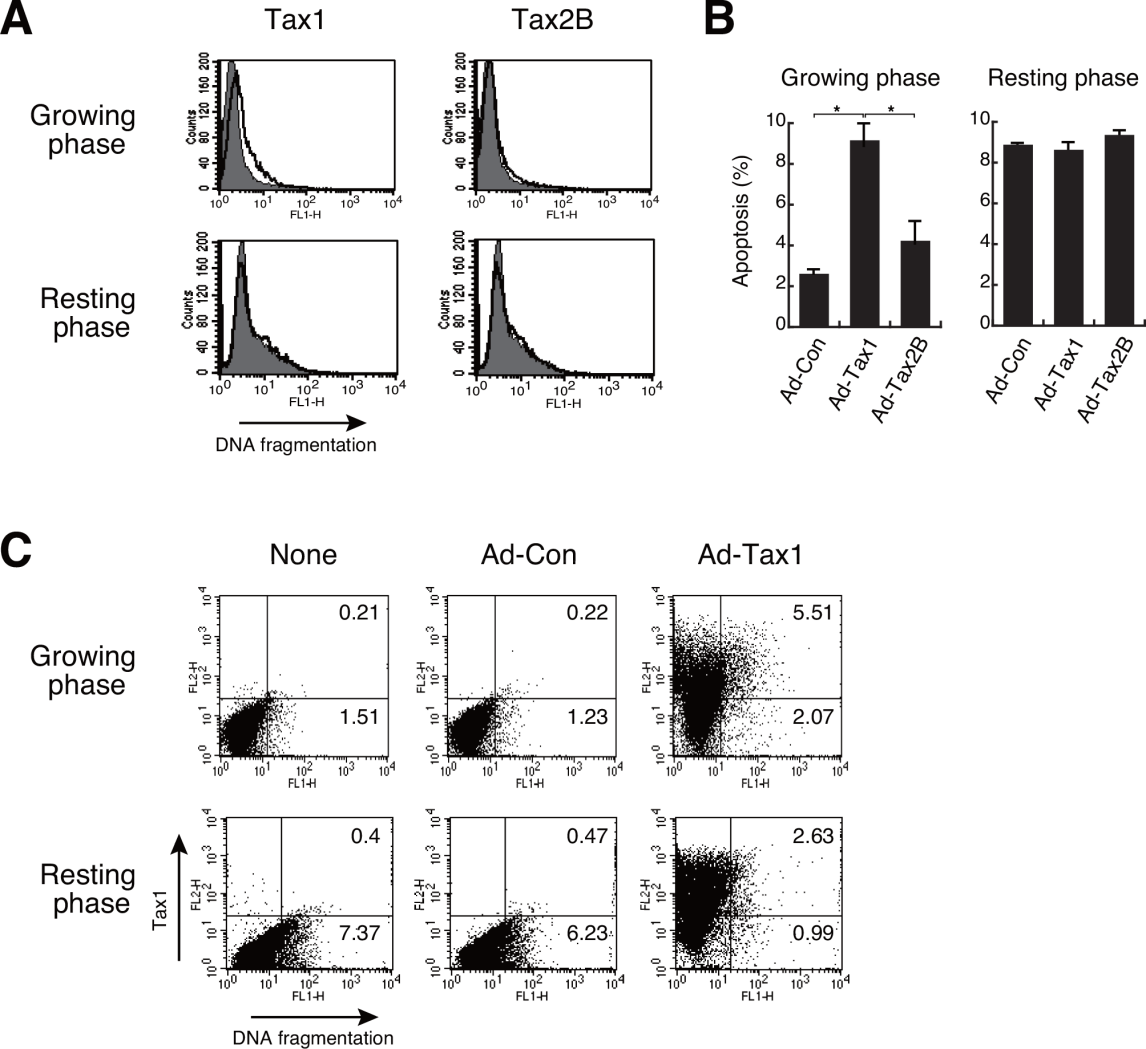
Induction of apoptosis by Tax1 in growing cells. (A) Growing or resting Kit 225 cells were infected with Ad-Tax1 or Ad-Tax2B, and cultured for 72 h. DNA fragmentation was detected by the TUNEL assay using a flow cytometer. The thick line and gray area indicate Ad-Tax1- or Ad-Tax2B-treated cells and Ad-Con-treated cells, respectively. (B) Percentage averages number of the cells undergoing apoptosis was calculated from three independent experiments. Values are shown as the means ± SE. *, *p* < 0.05. (C) DNA fragmentation and Tax1 expression were measured using a flow cytometer. Tax1 was detected with biotin-labeled anti-Tax1 antibody (Lt-4) and phycoerythrin-conjugated streptavidin, and DNA fragmentation was measured by TUNEL assay.

We then studied Tax1-induced growth inhibition in growing cells in terms of apoptosis induction. TUNEL assays detected DNA fragmentation in growing cells 72 h post adenoviral-mediated Tax1 expression, whereas no increase in Tax1-induced apoptosis was observed in resting cells (Fig 4A and 4B). Apoptosis in growing cells with Tax2B was slightly but distinctly induced, compared to the controls (Fig 4A and 4B). To analyze the link between Tax1 expression and apoptosis, we examined Tax1 expression and DNA fragmentation simultaneously by flow cytometry. Infection of growing cells with Ad-Tax1 significantly increased the apoptotic population (Fig 4C). This was in contrast to no appreciable changes in the population percentage with DNA fragmentation after Ad-Tax1 infection in resting cells (Fig 4C). The increase in the apoptotic population in growing cells was predominant in Tax1-positive cells. These results suggest that Tax1 elicits apoptosis in growing cells.

### Tax1 modulates apoptosis-related genes

**Fig 5:**
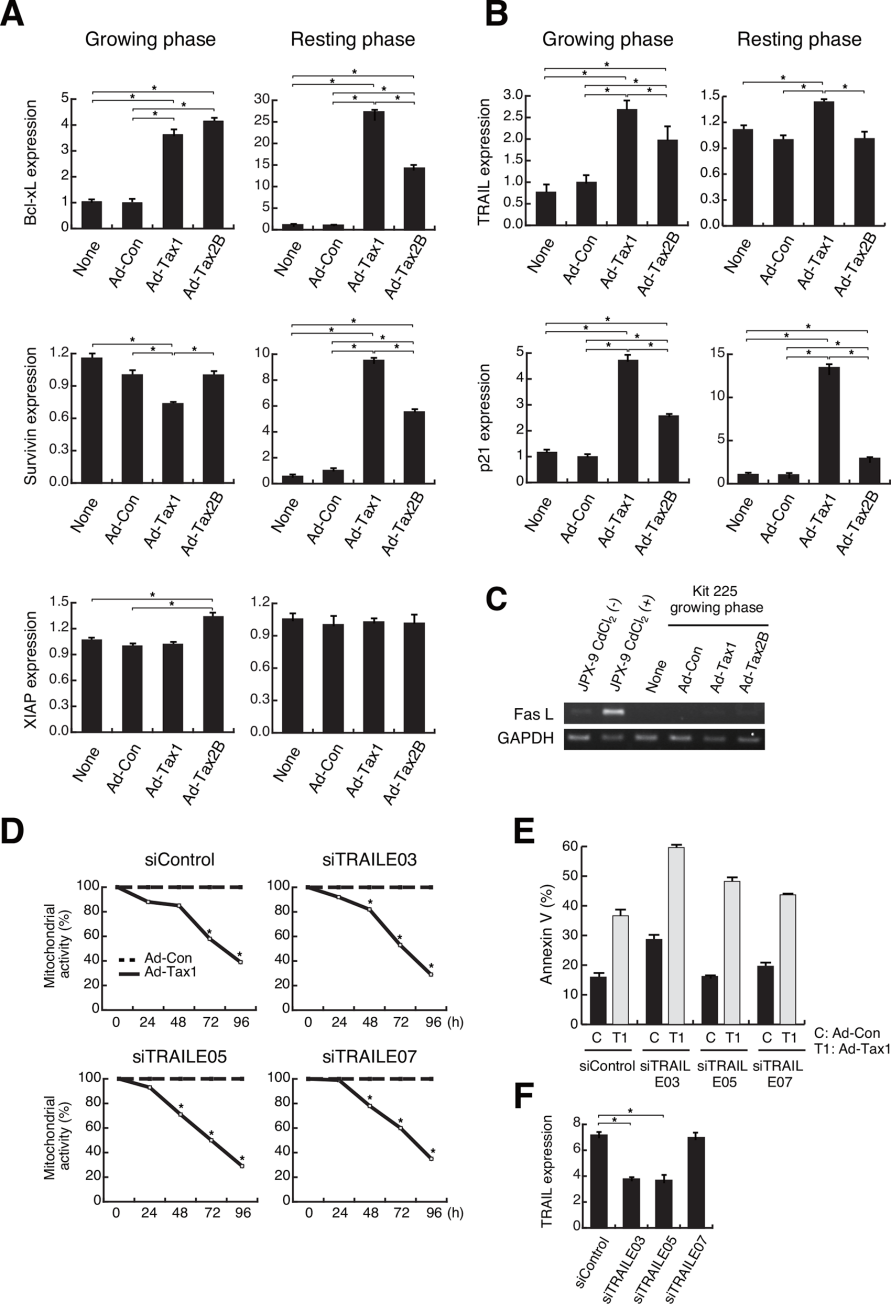
Modulation of apoptosis-related genes by Tax1. (A and B) Growing or resting Kit 225 cells were infected with the recombinant adenoviruses Ad-Tax1 or Ad-Tax2B, and cultured for 72 h. Gene expression was measured by qPCR. Values are shown as the means ± SE. *, *p* < 0.05. (C) JPX-9 cells were cultured with or without 20 μM CdCl_2_ for 48 h to induce Tax1 expression. Growing Kit 225 cells were infected with Ad-Tax1 and Ad-Tax2B, and cultured for 72 h. FasL and GAPDH expression was monitored by RT-PCR. (D) Growing Kit 225 cells were transfected with siRNA for TRAIL and cultured for 24 h, and infected with Ad-Con or Ad-Tax1, followed by harvest at indicated times. Mitochondrial activity was measured by MTT assay. Relative values of Ad-Tax1 to Ad-Con are shown. *, *p* < 0.05. (E) siRNA-treated growing Kit 225 cells were infected with Ad-Tax1 or Ad-Con, and cultured for 72 h. Annexin V positive cells were counted by Muse Cell Analyzer. (F) Growing Kit 225 cells were transfected with TRAIL-specific siRNA and cultured for 48 h. TRAIL expression was measured by qPCR. Values are shown as the means of the arbitrary copy numbers ± SE after normalization against 18 S rRNA content. *, *p* < 0.05.

The balance between anti-apoptotic and pro-apoptotic molecules is important for the induction of apoptosis [15]. Tax1 is known to induce anti-apoptotic molecules such as Bcl-xL, survivin and XIAP [16–18]. Changes in the expression of these genes were examined in resting and growing cells with or without Tax1 or Tax2B. qPCR analysis revealed up-regulated expression of Bcl-xL and survivin and unchanged expression of XIAP in resting cells with Tax1 and Tax2B (Fig 5A). Growing cells, which were induced for apoptosis by Tax1, showed that Tax1 down-regulated survivin expression and Tax2B slightly up-regulated XIAP expression (Fig 5A). Although Bcl-xL expression was increased by Tax1 and Tax2B in both resting and growing cells, the increase in growing cells was significantly lower than that in resting cells. Other anti-apoptotic proteins, Bfl-1 and cIAP2 [44, 45], which are shown to be induced by Tax1, were up-regulated in both growing and resting cells with Tax1 (data not shown). These results imply that Tax1 and Tax2B activate anti-apoptotic molecules in resting cells, presumably resulting in the prevention of apoptosis.

Induction of TRAIL and FasL by Tax1 has been shown to cause caspase-dependent apoptosis in Jurkat cells [20, 21, 26]. We thus examined the effects of Tax1 on the expression of TRAIL and FasL. TRAIL was up-regulated by Tax1 in both growing and resting Kit 225 cells (Fig 5B). Tax2B increased TRAIL expression in growing cells to a lesser extent than did Tax1 (Fig 5B). siRNA-mediated knockdown of TRAIL in growing cells however did not avoid Tax1-induced cell growth inhibition and apoptosis (Fig 5D and 5E). FasL was not produced in growing Kit 225 cells, even after introducing Tax1 (Fig 5C). The Jurkat derivative JPX-9 expresses FasL upon induction of Tax1 (Fig 5C) [26]. In addition, growing Kit 225 cells with Tax1 did not show Bim expression and caspase 8 and caspase 3 activation, which were observed in Jurkat cells with Tax1 (data not shown). These results indicate that TRAIL and FasL are not directly implicated in apoptosis in growing Kit 225 cells.

The CDK inhibitor p21 is a key factor for the Tax1-induced growth arrest in lymphoid cells [24]. On the other hand, Tax1-activated p21 plays a role in resistance to apoptosis [46]. We confirmed Tax1-mediated p21 expression in Kit 225 cells. Tax1 facilitated p21 expression in both growing and resting Kit 225 cells (Fig 5B). Tax2B also increased p21 expression in both phases (Fig 5B). p21 up-regulation by Tax1 and Tax2B may be one of the major causes of growth inhibition, as shown in Fig 2A.

### NF-κB/RelA is responsible for both cell survival and apoptosis

**Fig 6:**
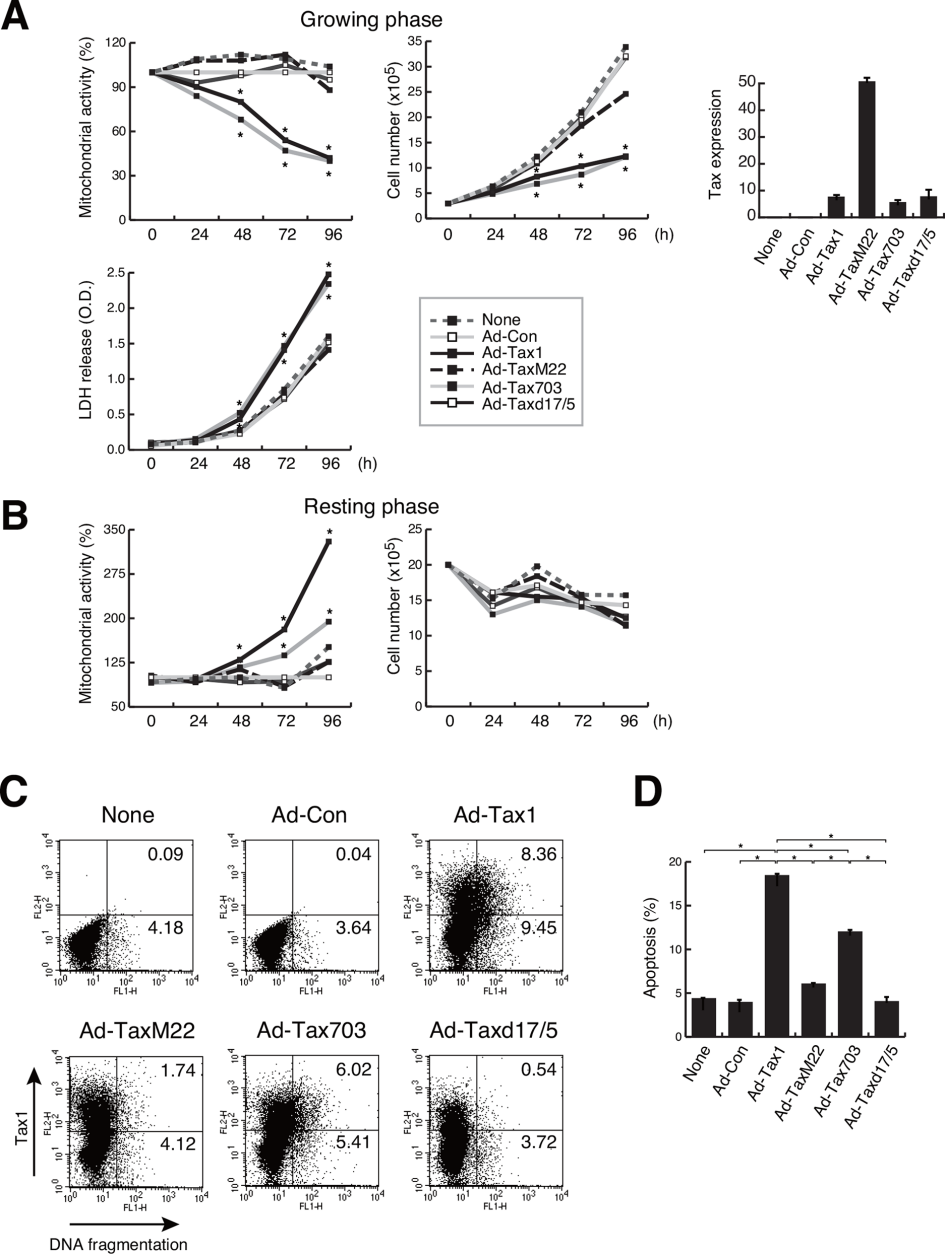
Effects of Tax1 mutants on growth inhibition and apoptosis. (A and B) Growing or resting Kit 225 cells were infected with Ad-Tax1, Ad-TaxM22, Ad-Tax703, Ad-Taxd17/5 or Ad-Con. After culture for the indicated time period, the mitochondrial activity, cell number and LDH activity of these cells were determined. Relative percentages to Ad-Con are shown. *, *p* < 0.05. Tax1 mutants expression was monitored by qPCR. Values are shown as the means of the copy numbers ± SE after normalization against 18 S rRNA content. (C) Tax1 expression and DNA fragmentation were measured by flow cytometry by using adenovirus-infected growing Kit 225 cells. (D) Percentage average number of the cells undergoing apoptosis in adenovirus-infected cells was calculated from three independent experiments. Values are shown as the means ± SE. *, *p* < 0.05.

**Fig 7:**
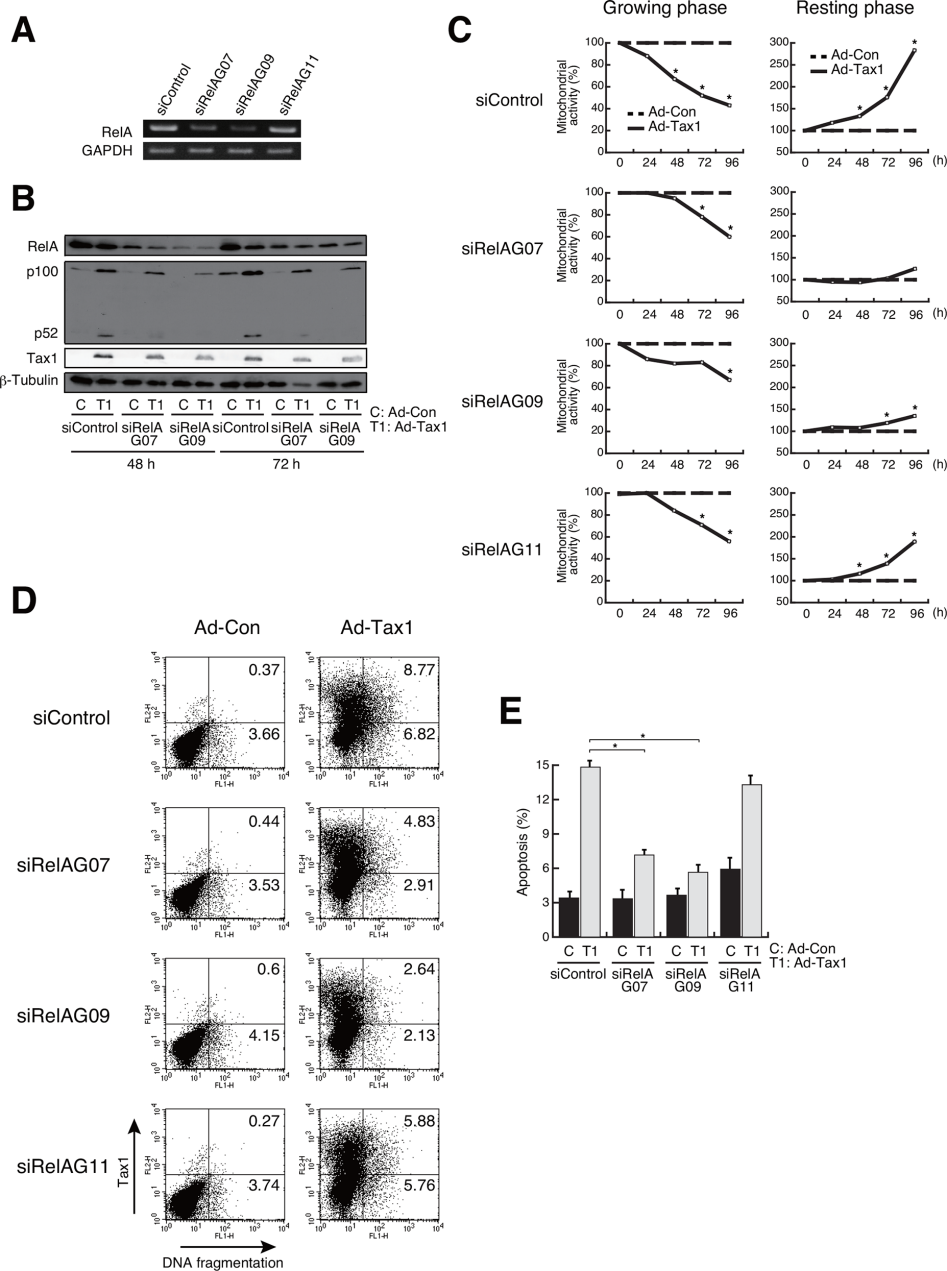
Participation of RelA in Tax1-mediated growth inhibition and apoptosis. (A) Growing Kit 225 cells were transfected with RelA-specific siRNA and cultured for 72 h. RelA expression was examined by RT-PCR. GAPDH was used as an internal control. (B) Growing Kit 225 cells were transfected with RelA-specific siRNA 24 h before adenovirus infection (Ad-Tax1 or Ad-Con), and harvested 48 h and 72 h post infection for western blotting. RelA, p100, p52 and Tax1 expression was monitored by immunoblotting with anti-RelA, anti-p52 and anti-Tax1 antibodies. β-Tubulin was used as an internal control. (C) siRNA-treated growing or resting Kit 225 cells were infected with Ad-Tax1 or Ad-Con, and cultured for indicated times. Mitochondrial activity was measured by MTT assay. Relative percentages of Ad-Tax1 to Ad-Con are shown. *, *p*<0.05. (D) siRNA-treated growing Kit 225 cells were infected with Ad-Tax1 or Ad-Con, and cultured for 72 h. Tax1 expression and DNA fragmentation were measured by flow cytometry. (E) Percentage average number of cells undergoing apoptosis was calculated from three independent experiments. Values are shown as the means ± SE. *, *p* < 0.05.

NF-κB has been known to be implicated in cell growth and survival through the induction of expression, including the growth factors [47, 48]. Among the NF-κB subunits, Tax1-activated RelA is reported to induce cellular senescence in HeLa cells [49, 50]. IKKγ-deficient Jurkat cells, which lack the ability to activate NF-κB, down-regulated Tax1-mediated apoptosis [20]. To examine if NF-κB is implicated in Tax1-mediated growth inhibition and cell survival, adenoviruses for Tax1 mutants were infected into growing and resting Kit 225 cells. Among the Tax1 mutants, TaxM22 and Taxd17/5, which lack the activity to activate NF-κB, exhibited effects similar to those exerted by the control virus Ad-Con, on the mitochondrial activity and the number of apoptotic cells in growing cells (Fig 6A, 6C and 6D). Tax703, which is capable of NF-κB activation, behaved like the wild-type Tax1 in case of LDH release into the culture medium as well as mitochondrial activity and cell number, indicating that Tax1-mediated cytotoxicity is NF-κB-dependent in growing cells. In resting Kit 225 cells, the NF-κB activation-deficient mutants TaxM22 and Taxd17/5 activated no or little, if any, mitochondrial activity (Fig 6B). These results prompted us to gain further insight into the molecular mechanism underlying Tax1-mediated apoptosis in growing Kit 225 cells. We examined a link between apoptosis and RelA, both of which are activated by Tax1. This issue was addressed by siRNA-mediated silencing of RelA. Of the three, two siRNAs against RelA, siRelAG07 and siRelAG09, effectively repressed RelA expression 72 h post siRNA introduction (Fig 7A). siRNA-induced down-regulation of RelA relieved the Tax1-dependent reduction of mitochondrial activity in growing cells (Fig 7C). In parallel, siRNA treatment for RelA decreased the number of apoptotic cells in Tax1-treated cells (Fig 7D and 7E). Interestingly, RelA knockdown in resting cells dramatically decreased the mitochondrial activity enhanced by Tax1 (Fig 7C). These results indicate that Tax1-activated RelA is implicated in the cell fate of both growing and resting cells.

### The non-canonical NF-κB pathway is associated with Tax1-induced apoptosis

**Fig 8:**
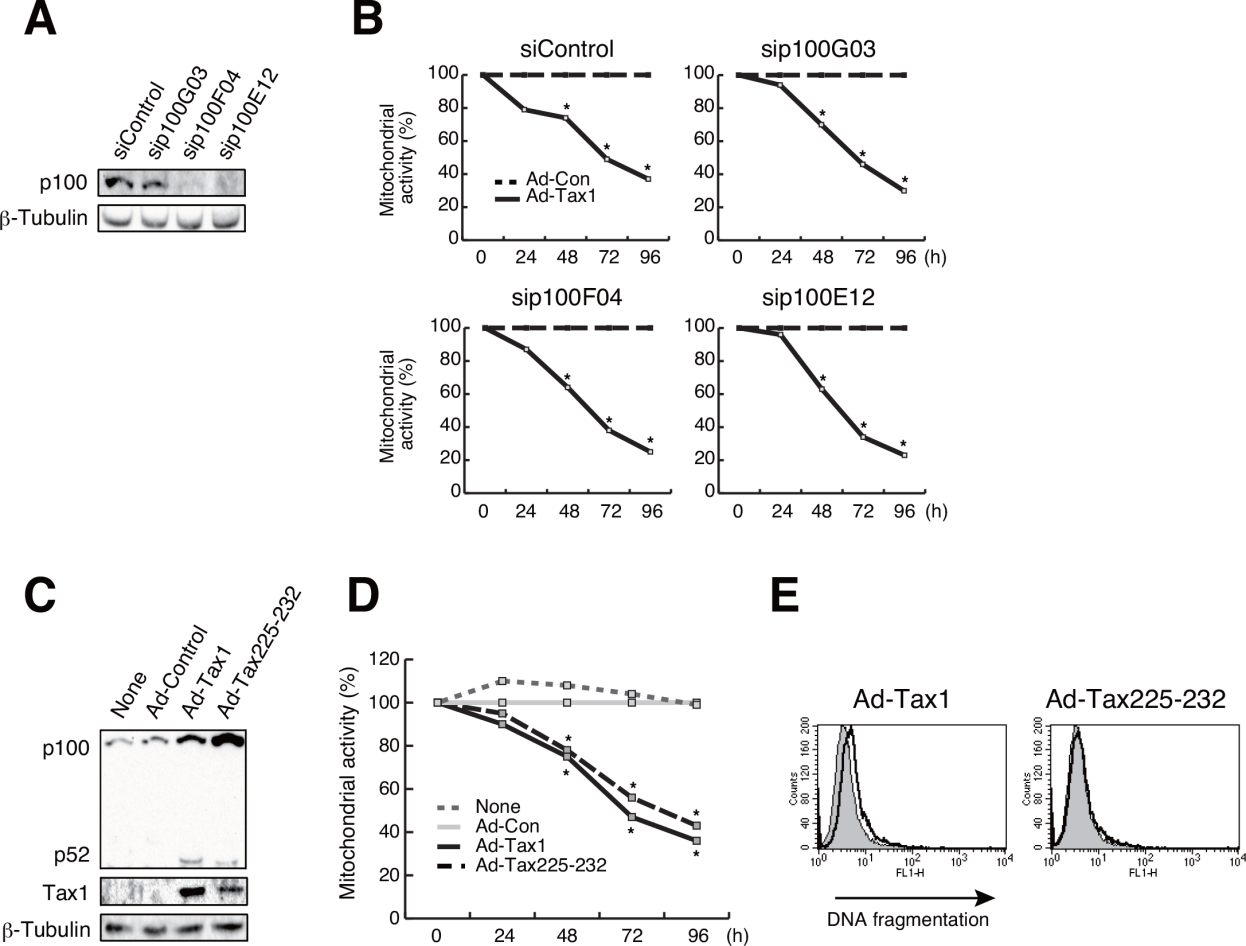
A link between apoptosis and the non-canonical NF-κB pathway. (A) Kit 225 cells were transfected with p100-specific siRNA and cultured for 48 h. p100 expression was detected by western blotting. β-Tubulin was used as an internal control. (B) Growing Kit 225 cells were transfected with p100-specific siRNA and cultured for 24 h, and infected with Ad-Con or Ad-Tax1, followed by harvest at indicated times. Mitochondrial activity was measured by MTT assay. Relative values of Ad-Tax1 to Ad-Con are shown. *, *p* < 0.05. (C) Expression of adenovirus-derived Tax1 and its mutant Tax225–232 proteins was measured by immunoblotting with anti-Tax1 antibody (Taxy-7). p100 and p52 levels were detected by anti-p52 antibody, which recognizes both p100 and p52. β-Tubulin was used as an internal control. (D) Growing Kit 225 cells were infected with Ad-Tax1, its mutant Ad-Tax225–232 or Ad-Con, and cultured for indicated times. Mitochondrial activity was measured by MTT assay. Relative percentages of Ad-Tax1 or Ad-Tax225–232 samples to Ad-Con samples are shown. *, *p* < 0.05. (E) Growing Kit 225 cells were infected with Ad-Tax1 or Ad-Tax225–232, and cultured for 72 h. DNA fragmentation was measured by flow cytometry. The thick line and gray area indicate Ad-Tax1- or Ad-Tax225–232-treated cells and Ad-Con-treated cells, respectively.

It may be intriguing to note that Tax2B was less effective in inducing apoptosis in growing cells, although Tax2B activated NF-κB (Fig 4A and 4B). Previous studies indicated differential effects in non-canonical NF-κB activation between Tax1 and Tax2B [51, 52]. The leucine zipper-like region (LZR) at amino acids 225–232 and the PDZ-binding motif (PBM) at C-terminus in Tax1, which are absent in Tax2B, are responsible for Tax1-mediated p100 to p52 processing, leading to non-canonical NF-κB activation [51, 52]. Knockdown of RelA in Kit 225 cells significantly affected Tax1-induced the p100 processing (Fig 7B). We thus evaluated the possible involvement of the non-canonical NF-κB pathway in Tax1-induced apoptosis. Introduction of p100-specific siRNA did not recover Tax1-mediated reduction of mitochondrial activity in growing cells (Fig 8B). Although a Tax1 mutant Tax225–232, which has low ability to process p100, poorly rescued mitochondrial activity (Fig 8C and 8D), it decreased an apoptotic population (Fig 8E), suggesting an association between Tax1-mediated apoptosis and the non-canonical NF-κB pathway.

### Tax1-mediated NF-κB/RelA regulates the expression of chemokines and cytokines

**Fig 9:**
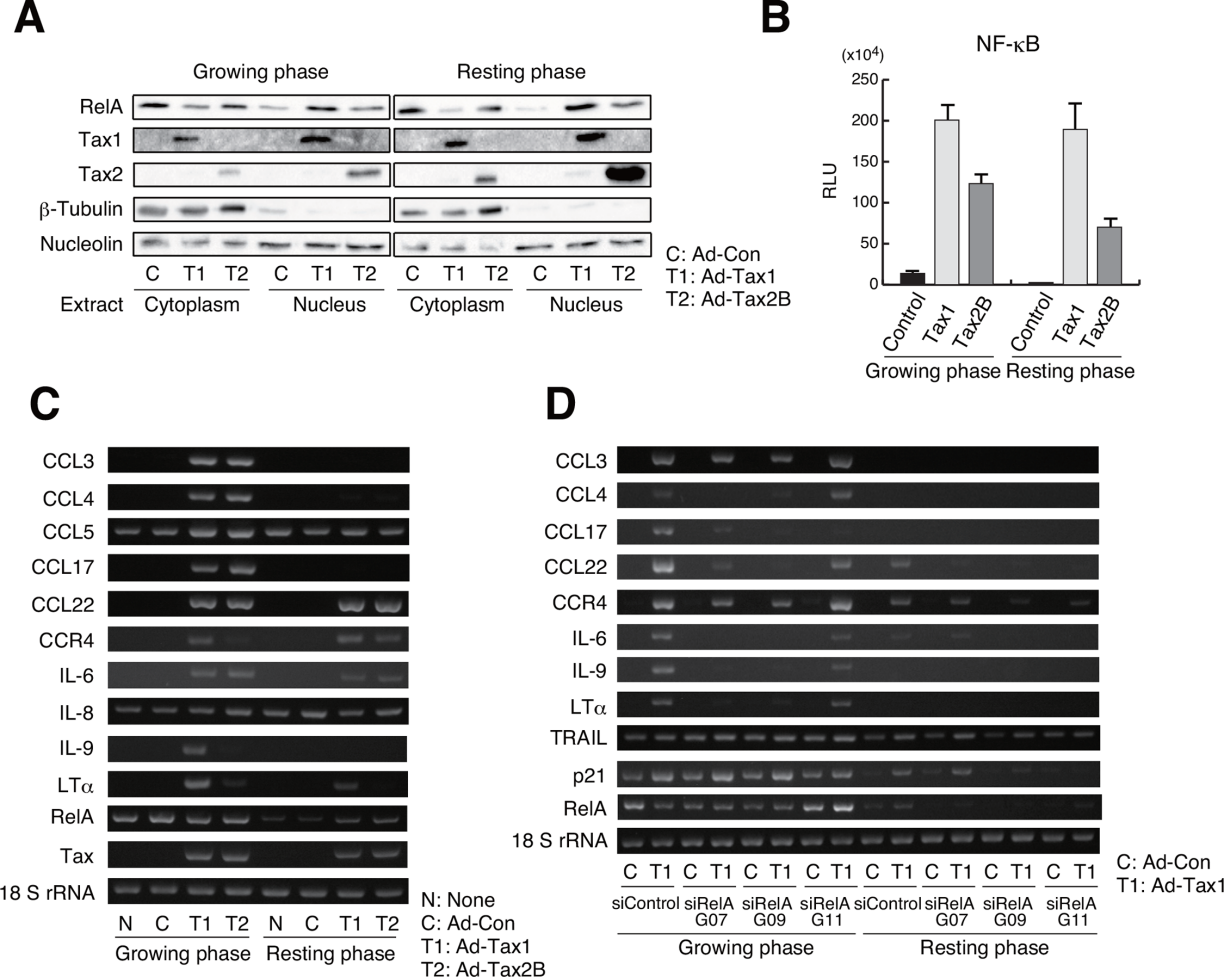
Induction of chemokines and cytokines by Tax1-mediated RelA activation. (A) Growing or resting Kit 225 cells were infected with Ad-Tax1 or Ad-Tax2B, and cultured for 72 h. The cytoplasmic and nuclear extracts were subjected to western blotting with antibodies for RelA, Tax1, Tax2, β-Tubulin and nucleolin. (B) The reporter plasmid carrying the NF-κB-binding sites was transfected into Kit 225 cells along with the Tax1 and Tax2B expression plasmids (pMT-2Tax and pHβAP-r-1-neoTax2B, respectively). After 48 h of culture with or without IL-2, the cells were harvested for luciferase assay, which was further normalized against protein content. Values are shown as the means ± SE. (C and D) Growing or resting Kit 225 cells were infected with Ad-Tax1 or Ad-Tax2B, and cultured for 72 h (C). siRNA-treated growing and resting Kit 225 cells were infected with Ad-Con or Ad-Tax1, and cultured for 48 h (D). Gene expression was monitored by RT-PCR. 18 S rRNA was used as an internal control.

To further analyze the observation that Tax1-induced RelA activation showed differential behavior between cells in growing and resting phases, the changes in the nuclear localization of RelA by Tax1 was examined by western blotting. Upon introduction of Tax1, the translocation of RelA to the nucleus was induced in both growing and resting cells (Fig 9A). Tax2B induced weak translocation of RelA to the nucleus, when compared to Tax1 (Fig 9A). To study whether NF-κB activation by Tax molecules depends on cell growth, Kit 225 cells were transiently transfected with the Tax1 or Tax2B expression plasmid along with a reporter plasmid containing NF-κB-binding sites, and then cultured with or without IL-2. Reporter assays showed that Tax1 and Tax2B activated NF-κB in both resting and growing cells (Fig 9B). Tax2B-mediated NF-κB activation seemed to be weaker than that mediated by Tax1 (Fig 9B). These results indicate that Tax1-mediated NF-κB activation is independent of cell growth.

NF-κB induces the expression of various chemokines, cytokines and their receptors, which have been shown to influence cellular senescence and apoptosis [53, 54]. The expression of NF-κB-regulated genes was examined in growing and resting cells. Tax1 and Tax2B induced the expression of CCL22, CCR4 and IL-6 in both growing and resting Kit 225 cells (Fig 9C). CCL3, CCL4 and CCL17, one of CCR4 ligands, were up-regulated by Tax1 and Tax2B only in the growing phase (Fig 9C). IL-9 and lymphotoxin α (LTα) expression was observed in growing cells with Tax1 (Fig 9C). siRNA treatment for RelA inhibited the induction of these gene (Fig 9D). These results suggest that Tax1-activated RelA is necessary for inducing the expression of these genes, but inadequate for inducing the expression of CCL3, CCL4, CCL17, IL-9 and LTα in the resting phase.

### Tax1 activates MAP kinase pathways

**Fig 10:**
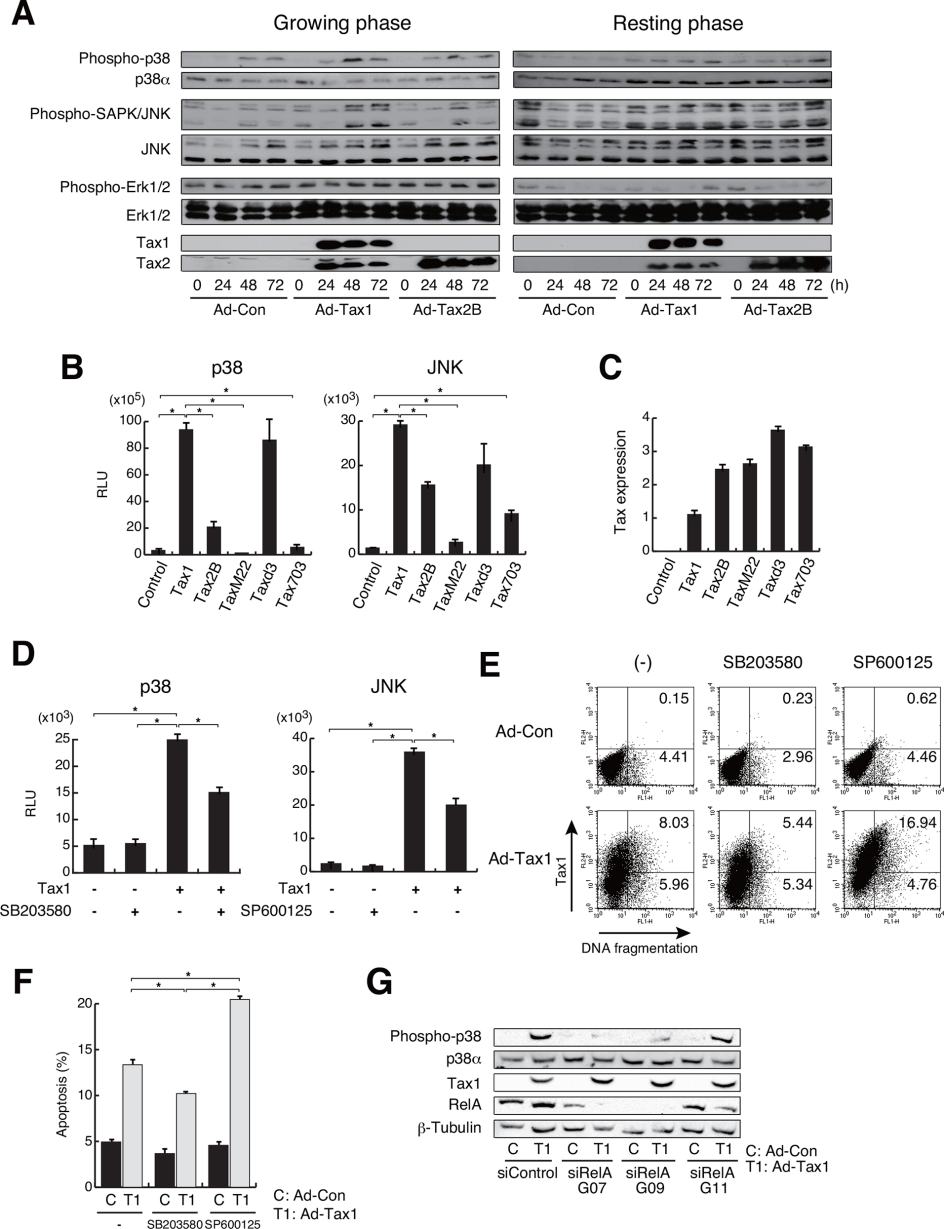
Activation of MAP kinase pathways by Tax1. (A) Growing or resting Kit 225 cells were infected with Ad-Tax1 or Ad-Tax2B, and cultured for indicated times. Phosphorylation and expression of p38, JNK and ERK were analyzed by western blotting. Expression of adenovirus-derived Tax1 and Tax2B in Kit 225 cells was detected with anti-Tax1 (Lt-4) and anti-Tax2 (GP3738) antibodies, respectively. (B and D) Growing Kit 225 cells were transfected with Tax1, its mutants and Tax2B expression plasmids along with pFR-Luc reporter vectors and either pFA-CHOP and pFA2-c-Jun, and cultured without (B) and with 5 μM p38 inhibitor SB203580 or 5 μM JNK inhibitor SP600125 (D) for 72 h. Values are shown as the means ± SE. *, *p* < 0.05. (C) Tax expression was examined by qPCR. Values are shown as the means of the copy numbers ± SE after normalization against 18 S rRNA content. (E) Growing Kit 225 cells were infected with Ad-Con or Ad-Tax1, and cultured with 5 μM SB203580 or SP600125 for 72 h. DNA fragmentation was detected by the TUNEL assay using a flow cytometer. (F) Percentage average number of the cells undergoing apoptosis in p38 or JNK inhibitor-treated Kit 225 cells was calculated from three independent experiments. Values are shown as the means ± SE. *, *p* < 0.05. (G) siRNA-treated growing Kit 225 cells were infected with Ad-Tax1 or Ad-Con, and cultured for 48 h. Phosphorylation and expression of p38, RelA, Tax1 and β-Tubulin were analyzed by western blotting.

Chemokines and cytokines affect the intracellular signaling pathways, including the mitogen-activated protein kinase (MAPK) signaling pathways [55]. The MAPK signaling pathways include the p38, c-Jun N-terminal kinase (JNK) and extracellular signal-regulated protein kinases (ERK) pathways and are involved in various physiological processes such as cell proliferation, differentiation and death [56]. To examine the effects of Tax1 and Tax2B on MAPK signaling pathways, we analyzed the phosphorylation of p38, JNK and ERK molecules as the activation markers of MAPK signaling pathway. p38 phosphorylation was induced in growing cells infected with Ad-Tax1 and Ad-Tax2B, even with Ad-Con (Fig 10A). Phosphorylation of p38 by Tax1 and Tax2B was prominent at 48 h of infection (Fig 10A). Ad-Con-dependent p38 phosphorylation may be due to the stress induced by adenovirus infection. Phosphorylation of JNK was observed prior to infection (Fig 10A). Infection with adenovirus reduced the levels of JNK phosphorylation at 24 h post infection, following which JNK phosphorylation was found to be up-regulated, in particular in growing cells expressing Tax1 and Tax2B (Fig 10A). Phosphorylation of ERK was constitutive in growing cells, irrespective of infection status and expression of Tax molecules. In resting cells, adenovirus infection reduced the levels of ERK phosphorylation (Fig 10A).

To exclude the effects of adenovirus infection, we utilized the luciferase assay PathDetect reporter system to monitor the activation of p38 and JNK signaling pathways. In this system, p38 and JNK, when phosphorylated, activate luciferase gene expression through the phosphorylation of chimeric CHOP and c-Jun molecules, respectively. Tax1 activated the p38 and JNK reporter pathways, and Tax2B showed similar but less effective activation of both pathways (Fig 10B). We further utilized the reporter system to investigate the implication of Tax1-targeted transcription factors in the activation of p38 and JNK pathways. The Tax1 mutants, TaxM22, Taxd3 and Tax703, which are defective for the activation of NF-κB, CREB and SRF, respectively, were transfected into Kit 225 cells along with the plasmids for the reporter assays. TaxM22 and Tax703 showed no or little, if any, activation of the p38 signaling pathway, which was significantly activated by Taxd3, suggesting that NF-κB and SRF are linked with the p38 signaling pathway (Fig 10B). The JNK signaling pathway was up-regulated by Taxd3 and Tax703 to some extent, but TaxM22 did not show significant activation of this pathway (Fig 10B). This implies that Tax1-dependent activation of the JNK signaling pathway occurs through NF-κB.

The link between Tax1-induced apoptosis and activation of the p38 and JNK signaling pathways was further studied using p38 (SB203580) and JNK (SP600125) inhibitors in growing cells. The inhibitors significantly reduced the luciferase activity associated with Tax1-mediated activation of p38 and JNK (Fig 10D). Tax1-induced apoptosis was relieved upon addition of the p38 inhibitor SB203580 (Fig 10E and 10F). In contrast, JNK inhibition increased Tax1-induced apoptosis (Fig 10E and 10F). These results indicate that Tax1-mediated activation of p38 is, at least in part, involved in Tax1-induced apoptosis and that the JNK signaling pathway might be implicated in cell survival in growing cells.

Tax1-activated NF-κB including RelA and the p38 signaling pathway, and Tax1-mediated apoptosis was closely associated with the activation of both NF-κB and p38. These observations prompted us to examine a correlation between the activation of RelA and p38. Phosphorylation of p38 was abolished by the addition of siRNA (G07 and G09) for RelA (Fig 10G), presumably indicating that RelA is essential for the activation of p38 signaling pathway in growing cells with Tax1.

## Discussion

HTLV-1 Tax is shown to have pleiotropic functions; cellular senescence in HeLa cells and cell survival in MEF, both of which are mediated by NF-κB/RelA [50, 57]. These results demonstrated that Tax1 is involved in cell growth inhibition and survival in dividing and non-dividing cells, respectively, although they were obtained by independent experimental models. To evaluate the dual functionality of Tax1 being dependent upon cell division, we established systematic experimental conditions with the human T-cell line Kit 225 cells, whose cell growth can be regulated experimentally. The present study showed that Tax1 induces different cell fates in a cell growth-dependent fashion; cell growth inhibition with apoptosis in growing cells in contrast to cell survival in resting cells. Our results that Tax1-dependent activation of NF-κB is associated with both phenotypes and that NF-κB/RelA knockdown relieves Tax1-induced cell growth inhibition and apoptosis strongly indicate that Tax1-activated RelA is functionally critical for cell growth inhibition and apoptosis in growing Kit 225 cells (Figs 6 and 7) in accordance with previous reports observed in independent experimental models [20, 50]. Withdrawal of the p52 precursor p100 did not recover cell growth inhibition by Tax1, while apoptosis was not observed with Tax225–232, which fails to activate the non-canonical NF-κB pathway (Fig 8). These results are partly consistent with previous results that p100 knockdown does not rescue Tax1-induced senescence in HeLa cells [49]. RelA is also a regulator of the expression and processing of p100 (Fig 7B). Thus, we confirm that RelA is a key factor in Tax1-induced cell growth inhibition and apoptosis. On the other hand, resting cells also required Tax1-activated RelA for survival and proliferation (Figs 6B and 7C), in accordance with previous observations that NF-κB activated the cellular genes involved in cell survival and proliferation [13, 17, 47, 58–60]. The Tax1 mutant TaxM22, which lacks the ability to activate NF-κB, failed to proceed the cell cycle in resting Kit 225 cells [10], supporting the idea that NF-κB is important for Tax1-mediated cell cycle progression in resting cells. Collectively, Tax1-activated RelA plays a role in cell survival and death in resting and growing cells, respectively.

Cell cycle profiles showed that Tax1 and Tax2B expression induces the accumulation of cells with 3C DNA content (Fig 1). This may be attributed to the aberrant DNA synthesis in Tax-expressing cells, wherein new DNA replication cycles may occur before the completion of the previous cycles. Our preliminary results showed that Tax1 did not promote the phosphorylation of γH2AX, a marker of DNA damage in growing cells (data not shown). These results suggest that Tax1 and Tax2B induce replication stress in our experimental conditions. In addition to DNA damage and replication stress, various stimulations such as cytokines and oncogenes are shown to activate the NF-κB pathway [61–63]. Those stimulations induce different post-translational modifications of RelA with position-specific phosphorylation and acetylation, leading to differences in the expression profiles of RelA target genes [61]. Tax1 up-regulates NF-κB activity through RelA, by acetylation at lysine 310, which has been shown to be facilitated by Tax1 and recruits the bromodomain-containing factor Brd4 [57]. Phosphorylation of RelA at serine 276 induces the formation of a complex with the transcriptional activator CBP/p300, while the repressor HDAC is associated with non-phosphorylated RelA [64]. As Tax1-activated RelA induces opposite phenotypes in growing and resting cells, post-translational modifications of RelA may differ from each other.

Tax1-induced cell death in Jurkat cells has been shown to be triggered by the expression of TRAIL or FasL [20, 26]. Although TRAIL and FasL genes do not contain any NF-κB-binding sequences in the promoter regions, the expression of these genes was suppressed by inhibition of the NF-κB signaling pathway. This suggests the indirect or secondary effects of NF-κB activation on the expression of these death molecules in Jurkat cells. Our results showed that up-regulation of TRAIL, but not FasL, by Tax1 was observed in growing Kit 225 cells (Fig 5B and 5C). The resting Kit 225 cells also showed enhanced TRAIL expression in response to Tax1 (Fig 5B). Deprivation of TRAIL expression did not rescue growth inhibition and apoptosis (Fig 5D and 5E). In addition, TRAIL expression was not down-regulated by RelA deprivation in growing cells with Tax1 (Fig 9D). These results indicate that Tax1-induced apoptosis is TRAIL- and FasL-independent in growing Kit 225 cells.

The induction of growth inhibition and apoptosis by Tax2B was weak compared to that by Tax1. Activation of p38 monitored by phosphorylation (Fig 10A) and reporter luciferase activity (Fig 10B) were higher in Tax1-expressing cells than Tax2B-expressing cells. In addition, Tax1 activated NF-κB to a higher extent than did Tax2B in the reporter assay (Fig 9B). We observed similar expression levels of Tax1 and Tax2B molecules in these experiments. It is intriguing to note that the Tax1 mutant TaxM22, which is defective for NF-κB activation, was significantly less effective in p38 activation and apoptosis induction, and that the p38 inhibitor repressed Tax1-induced apoptosis. These results propose the notion that Tax1 induces apoptosis through the activation of p38 signaling pathway, which may be activated, in part, by NF-κB.

Recent studies reveal that NF-κB and p38 activation is implicated in the senescence-associated secretory phenotype (SASP) and oncogene-induced senescence (OIS) with the induction of inflammatory chemokines and cytokines [65–68]. Senescence is thought to be one of host defense mechanisms to avoid transformation of abnormal cells, while senescent cells promote tumorigenesis in neighboring pre-neoplastic cells [67, 69]. The mechanisms underlying senescence include a process wherein oxidative and genotoxic stress activate NF-κB, which further induces pro-inflammatory mediators such as IL-1, IL-6, IL-8, CCL3 and CCL4; this leads to the activation of p38 pathway through cellular environmental stress [54]. Indeed, we observed that RelA deprivation decreased p38 phosphorylation in growing cells with Tax1 (Fig 10G). We confirmed that the inflammatory mediators IL-6, CCL3 and CCL4 are induced by Tax1 and Tax2B in growing cells (Fig 9C). Tax1-expressing cells may reflect the SASP and OIS phenomena and influence extracellular environment through the release of inflammatory effectors.

We reported that Tax2B promoted cell proliferation through the induction of IL-2 in PBLs and HTLV-2-infected cells [28, 38]. In resting-induced normal PBLs, Tax2B, but not Tax1, induced IL-2 production to a large extent, leading to cell proliferation in an autocrine-loop manner (Fig 2C). Tax2B is thus expected to induce normal cell cycle progression through the IL-2-mediated autocrine loop without aberrant DNA synthesis and formation of multinucleated cells, which have been shown to be induced by Tax1 through improper cell cycle progression and mitosis block [70]. In fact, multinucleated cells were obseved with Tax1 rather than Tax2B in Kit 225 and Tax-inducible Jurkat cells (Fig 1) [19]. As Kit 225 cells lack the ability to produce IL-2, Tax2B is not effective in IL-2 autocrine loop cell growth (data not shown). Therefore, Kit 225 cells are suitable for studying the effects of Tax2B on the expression of cell cycle regulatory genes in the resting state. Tax2B induced low-to-moderate expression of the cyclin D2 and cyclin E genes compared to Tax1 in the resting Kit 225 cells (Fig 3A), reasoning why Tax2B mediates weak progression of the cell cycle and indicating that IL-2 produced by Tax2B is a major factor promoting cell cycle progression in HTLV-2 infected cells. The differences in the activation of cell cycle-related genes between Tax1 and Tax2B may be associated with HTLV-1 pathogenesis. The weak formation of multinucleated cells by Tax2B may not allow the development of malignant diseases.

When stimulated, the resting T-cells infected with HTLV-1 first produce Tax1, which accelerates HTLV-1 transcription, presumably in association with cellular factors. On the other hand, Tax1 is a major target among the viral antigens of cytotoxic T-cells in the host [71, 72]. The constitutive expression of Tax1 is ultimately a disadvantage for HTLV-1. Thus, Tax1 may function as repressor to HTLV-1 expression via growth arrest. Meanwhile, Tax1 contributes to cell survival through up-regulation of molecules involved in mitochondrial metabolism and induction of various growth factors and anti-apoptotic molecules in resting cells. Transcription of the p21 gene was greatly up-regulated by Tax1, in resting cells as well as in growing cells (Fig 5B). p21 molecule has been reported to contribute to cell survival and cell death resistance in response to extracellular stimuli [46]. This may be a strategy employed by HTLV-1 to aid longer survival, which is advantageous to HTLV-1 latent infection. During long-term latency, HTLV-1-infected T-cells show increased production of chemical mediators including cytokines, and undergo genetic and epigenetic alterations, in part, through Tax1 expression, leading to HAM/TSP and ATL, respectively. To summarize, this study implies that Tax1 driven-paradoxical phenomena are important for persistent latency and pathogenesis of HTLV-1.

## Supporting Information

S1 Table Summary of PCR primers. (DOCX)

## Abbreviations

HTLV-1: human T-cell leukemia virus type 1
ATL: adult T-cell leukemia
HAM/TSP: HTLV-1-associated myelopathy/tropical spastic paraparesis
IL-2: interleukin 2
PBLs: peripheral blood lymphocytes
PHA: phytohemagglutinin
RT-PCR: reverse transcription-PCR
qPCR: quantitative PCR
PI: propidium iodide
TUNEL: TdT-mediated dUTP nick end labeling
