# Supplementary material for: Induction of Cell Death in Growing Human T-Cells and Cell Survival in Resting Cells in Response to the Human T-Cell Leukemia Virus Type 1 Tax
Source: PLoS One. 2016 Feb 1;11(2):e0148217. doi: 10.1371/journal.pone.0148217 (PMC4734616; doi:10.1371/journal.pone.0148217)
Supplement: S1 Table — (DOCX) [file pone.0148217.s001.docx]

**S1 Table. Summary of PCR primers.**

|  | Forward | Reverse |
| --- | --- | --- |
| CDK2 | 5’-ggatgcctctgctctcactggc-3’ | 5’-ggtctcggtggaggacccga-3’ |
| CDK4 | 5’-tggacaaggcacccccacca-3’ | 5’-ggccaggccaaagtcagcca-3’ |
| cyclin D2 | 5’-ccgcaacctgctccgagacg-3’ | 5’-ggagtcgggaccccagccaa-3’ |
| cyclin E | 5’-cggacaagaccctggcctcagg-3’ | 5’-cggtcacgtttgccttcctctt-3’ |
| FasL | 5’-gccatgcagcagcccttcaa-3’ | 5’-ggccactttcctcagctccttttt -3’ |
| Survivin | 5’-cctggctcctctactgtttaac-3’ | 5’-ctgtctcctcatccacctgaa-3’ |
| Bcl-xL | 5’- agaaccactacatgcagcccat-3’ | 5’-tgtgaattctgaggccaaggg-3’ |
| XIAP | 5’-catcaacactggcacgagcagg -3’ | 5’-tcttggggttaggtgagcatagt-3’ |
| RelA | 5’-ccgggatggcttctatgagg-3’ | 5’-gaggggtccggaacacaarg-3’ |
| p100 (NFKB2) | 5’-gctgcgcttctctgccttcc-3’ | 5’-tccgcttccgctgcacctctt-3’ |
| p21 (CDKN1A) | 5’-ggcccagtggacagcgagcag-3’ | 5’-atcagccggcgtttggagtggtag-3’ |
| TRAIL | 5’- tgacagttattgggaccccaa-3’ | 5’-cttgaactgtagaaatggtttcc-3’ |
| CCL3 | 5’-gctgcccttgctgtcctcctct-3’ | 5’-ctgccggcttcgcttggttag-3’ |
| CCL4 | 5’-cttcctcgcaactttgtggtaga-3’ | 5’-gaagcatccgggctcaggtg-3’ |
| CCL5 | 5’-ccccgtgcccacatcaaggagta-3’ | 5’-ggagcgggtggggtaggatagtga-3’ |
| CCL17 | 5’-atggccccactgaagatgct-3’ | 5’-ttggggtccgaacagatgg-3’ |
| CCL22 | 5’-aggacagagcatggctcgcctacaga-3’ | 5’-taatggcagggaggtagggctcctga-3’ |
| CCR4 | 5’-aagaagaacaaggcggtgaagatg-3’ | 5’-aggcccctgcaggttttgaag-3’ |
| IL-6 | 5’-ccagtacccccaggagaagat-3’ | 5’-gttgggtcaggggtggttatt-3’ |
| IL-8 | 5’-accggaaggaaccatctca-3’ | 5’-ctcagccctcttcaaaaac-3’ |
| IL-9 | 5’-ggcaggccaggggtgtcc-3’ | 5’-ttgcctgccgtggtttggtt-3’ |
| lymphotoxin α (LTα) | 5’-gcccgtcagcaccccaagat-3’ | 5’-gcgaaggctccaaagaagacagta-3’ |
| Tax | 5’-tccttccccacccagagaac-3’ | 5’-atggggggggaaagctggta-3’ |
| GAPDH | 5’-ggagtccactggcgtcttca-3’ | 5’-gaggggccatccacagtctt-3’ |
